# A Co-Culture System with an Organotypic Lung Slice and an Immortal Alveolar Macrophage Cell Line to Quantify Silica-Induced Inflammation

**DOI:** 10.1371/journal.pone.0117056

**Published:** 2015-01-30

**Authors:** Falk Hofmann, Robert Bläsche, Michael Kasper, Kathrin Barth

**Affiliations:** Institute of Anatomy, Medical Faculty Carl Gustav Carus, Dresden University of Technology, Dresden, Germany; University of North Dakota, UNITED STATES

## Abstract

There is growing evidence that amorphous silica nanoparticles cause toxic effects on lung cells *in vivo* as well as *in vitro* and induce inflammatory processes. The phagocytosis of silica by alveolar macrophages potentiates these effects. To understand the underlying molecular mechanisms of silica toxicity, we applied a co-culture system including the immortal alveolar epithelial mouse cell line E10 and the macrophage cell line AMJ2-C11. In parallel we exposed precision-cut lung slices (lacking any blood cells as well as residual alveolar macrophages) of wild type and *P2rx7^−/−^* mice with or without AMJ2-C11 cells to silica nanoparticles. Exposure of E10 cells as well as slices of wild type mice resulted in an increase of typical alveolar epithelial type 1 cell proteins like T1α, caveolin-1 and -2 and PKC-*β*1, whereas the co-culture with AMJ2-C11 showed mostly a slightly lesser increase of these proteins. In *P2rx7^−/−^* mice most of these proteins were slightly decreased. ELISA analysis of the supernatant of wild type and *P2rx7^−/−^* mice precision-cut lung slices showed decreased amounts of IL-6 and TNF-α when incubated with nano-silica. Our findings indicate that alveolar macrophages influence the early inflammation of the lung and also that cell damaging reagents e.g. silica have a smaller impact on *P2rx7^−/−^* mice than on wild type mice. The co-culture system with an organotypic lung slice is a useful tool to study the role of alveolar macrophages during lung injury at the organoid level.

## Introduction

Silicon dioxid (silica, SiO_2_) is one of the most common elements on earth, but inhalation of silica can lead to an injury of the lung, prolonged inhalation can even lead to chronic inflammation and fibrosis due to deposition of silica particles in the lung. The typical lung reaction induced by chronic inhalation of crystalline silica is silicosis, a generally progressive fibrotic lung disease (pneumoconiosis), exemplified by the development of silicotic nodules composed of silica particles surrounded by whorled collagen in concentric layers, with macrophages, lymphocytes, and fibroblasts in the periphery. The proliferation of lung epithelial cells is a prominent feature of lung tissue response following silica-induced lung injury and alveolar macrophages are recognized as a major contributing cell to the lung inflammatory process.

The growing abundance and industrial applications of nanotechnology has resulted in a recent shift of toxicological research towards nanoparticles. The particles seem to affect epithelial cells, fibroblasts and lung macrophages [1–4]. Ultrafine particles (< 0,1 µm) cause a greater inflammatory response than do fine particles (< 2,5 µm) per given mass [5–7]. Cytotoxicity of silica may be caused by penetration of the cytoplasm; whereas nano-silica up to ca. 100 nm can even penetrate into nuclear regions [8–10], where the silica particles lead to an inflammatory response through to generation of reactive oxygen species (ROS) [9, 11, 12]. The cytotoxicity of silica seems to be dose- time- and size-dependent [11–16].

The acute injury of the lung is characterized by an unbalanced quantity or quality of the inflammatory response to epithelial or endothelial injury leading to inflammation, cellular damage, apoptosis or necrosis of cells of the alveolar-capillary barrier [17]. Dysregulated resolution and repair can result in an excessive fibroproliferation with development of pulmonary fibrosis [18]. To model the early inflammatory stage of both diseases silica can be used due to its generation of a chronic inflammation as reviewed in [19].

One protein intensively studied in context with pulmonary fibrosis and lung inflammation is caveolin-1 (Cav-1), which is abundantly expressed in lung epithelia, endothelia, and fibroblasts [20]. Cav-1 contributes both to endothelial and epithelial pathologies: Endothelial Cav-1 regulates microvascular permeability by controlling caveolar transcytosis e.g. albumin and inhibits the endothelial nitric oxide synthaseactivity [21]. Epithelial Cav-1 controls a common pathway of apoptosis by regulating survivin [22–24]. Further Cav-1 promotes apoptosis in the bleomycin fibrosis model and plays a role in epithelial cell growth arrest and senescence [25–29]. Closely related to Cav-1 in alveolar epithelial cells is a purinergic receptor, P2X7R, due to its molecular interaction and its occurrence in the lipid raft/caveolae fraction. P2X7 receptors are selectively localized in alveolar epithelial type I cells (AEC I) and are thought to play a role in fluid haemostasis [30]. Their involvement in lung inflammation and tissue regeneration needs to be further explored.

Riteau et al. [31] demonstrated that phagocytosis of silica particles induces the active release of ATP by human peritoneal macrophages and purinergic signaling leading in inflammasome activation.

Moncao-Ribeiro et al. [32] showed *in vitro* silica-induced IL-1β secretion in macrophages (AMJ2-C11). It was also demonstrated that pre-incubation with a P2X7 receptor specific inhibitor completely inhibited silica-induced IL-1β secretion. The alveolar macrophages treated with silica and P2X7 receptor inhibitor have shown decreased silica phagocytosis. These results support the findings of Wiley and Gu [33] who observed that P2X7 receptor expressed in phagocytic cells augments the engulfment of latex beads and bacteria.

We used a co-culture system consisting of alveolar epithelial cells (E10) and an immortal macrophage cell line AMJ2-C11 as an approach to reflect the interactions between different cell types in the lung after exposure to nanoparticles. In parallel we included a new co-culture system with an organotypic lung slice (precision-cut lung slices, PCLS) and immortal alveolar macrophage cell line AMJ2-C11 to quantify silica-induced inflammation.

The aim of this study was to obtain basic data on the biological effects of the silica nanoparticle—macrophage interaction on epithelial cells.

## Animals, Material and Methods

### Ethics Statement

All animal experiments were approved by the ethics committee of the Dresden University of Technology and the license for removal of organs was provided by the Landesdirektion Dresden (file no. 24–9168.24–1/2007–26; file no. 24–9168.24–1/2010–11).

### Mice

For our experiments we used wild type (C57BL/6; Charles River, Wilmington, MA, USA) and *P2rx7^−/−^*mice (B6.129P2-*P2rx7^tm1Gab^/J;* Pfizer, New York City, NY, USA) [34]. Our animals were housed at the Animal Care Facility at the Medical Faculty “Carl Gustav Carus” of Dresden, University of Technology. The mice always had free access to standard chow and water. All performed procedures were in accordance with the Dresden University of Technology Animal Care and Use Committee Guidelines. We examined lung tissues from male and female mice (8–18 weeks) for our experiments.

### Silica

We used nano-silica from Sigma Aldrich (St. Louis, MO, USA). Following specifications of the nanoparticles were provided by the supplier: spherical, porous, particle size 5–15 nm (measured with transmission electron microscopy).

Concentration of silica in the experiments is given as mass per area for cultures with adherent cells due to the tendency of silica to sediment and as mass per volume in non-adherent culture because of the agitation in a roller system.

### Cell lines and cell culture

As a model for the alveolar epithelium we used a spontaneous immortalized lung non-tumor cell line of the mouse (E10 cells; kindly provided by M. Williams, Pulmonary Center, Boston University School of Medicine, Boston, MA, USA) as previously reported [35]. E10 cells were cultured in DMEM/Ham’s F12 medium from Gibco (ThermoFisher Scientific, Waltham, MA, USA), supplemented with 5% (v/v) fetal bovine serum (PAN Biotech GmbH, Aidenbach, Germany) and 2.5 mM L-glutamine (Merck, Darmstadt, Germany). E10 cells were grown at 37°C in a 5% CO_2_ atmosphere up to a confluence of approximately 60–80%. They were seeded at a density of 2x10^4^ cells/ml and passaged three times per week up to 25 passages.

Secondly we used AMJ2-C11 alveolar macrophages infected with the J2 retrovirus carrying the v-raf and v-myc oncogens (ATCC, Wesel, Germany) [36].

AMJ2-C11 cells were cultivated with DMEM high-glucose medium (Merck) supplemented with 5% (v/v) fetal bovine serum, 1% (v/v) sodium pyruvate, 2% (v/v) L-glutamine, 2% (v/v) NaHCO_3_ and 5 mM HEPES (all from Merck). They were grown at 37°C in a 5% CO_2_ atmosphere. They were seeded at a density of 2x10^5^ cells/ml and passaged two times per week up to 40 passages.

As experimental set-ups we used (i) a E10-mono-culture and (ii) a co-culture of E10 and AMJ2-C11. The cells were treated with 50, 100 or 200 µg nano-silica per cm^2^ ground area of the flasks by adding the nano-silica to the medium, because of the adherent character of this experimental set-up.

### Precision-cut lung slices and tissue culture

Following the preparation procedure of precision-cut lung slices (PCLS) published by Held et al.[37] and recently described by Ebeling et al.[38] tissue preparation was done with several modifications. Wild type and *P2rx7^−/−^* mice were anesthetized with an overdose of 5% (w/v) thiopental (Thiopental 0.5 g, Inresa, Freiburg, Germany) and exsanguinated by cutting the right renal artery. The chest wall was opened and the lungs were perfused with 10 ml PBS at RT through the right ventricle. Subsequently the trachea was cannulated with a 20-gauge catheter (Saf-T-Intima with Y adapter, BD Biosciences, San Diego, CA, USA) and the lungs were filled with 1% (w/v) low-melting agarose (Sigma-Aldrich) solved in PBS at 37°C *in situ*. For agarose solification the mouse was cooled with ice for 15 minutes at 4°C. Next, solidified lungs were removed and separated into the different lobes. Each lobe was embedded in 3% (w/v) prewarmed agarose at 42°C. For gelling, agarose was cooled with ice at 4°C for 15 minutes. Afterwards each lobe was cut with Krumdieck tissue slicer (Alabama Research and Development, Munford, AL, USA) into 500 μm thick slices using DMEM/F12 medium with 15 mM HEPES and 2.5 mM L-glutamine cooled down to 4°C. Then tissue slices were transferred into cell culture plates containing above-mentioned medium including 1% (v/v) pen/strep (Merck) and 0.5% (v/v) fetal bovine serum at 37°C. During the following 3 hours medium was changed every 30 minutes. For add-on tissue culture five lung slices each were transferred into a 20 ml glass vial (Scintillation Vial, KG-33 Borosilica Glass, VWR International, Radnor, PA, USA) containing 3 ml medium for culture without macrophages and 5 ml medium for co-culture with macrophages. For treatment with nano-silica medium was completed with 1600 µg/ml nano-silica, because of the non-adherent character of this experimental set-up. Glasses were closed with Filter Easy Caps 25 cm^2^ (ThermoFisher Scientific). The vials were put in a roller system (Stuart Rotator SB3, Bibby Scientific, Staffordshire, United Kingdom) rotating at 5 rpm in a 5% CO_2_ atmosphere at 37°C. After 72 hours incubation the lung slices were homogenized for Western Blotting or embedded in paraffin for immunostaining. The supernatant was collected for ELISA analysis.

### Western Blot analysis, conventional histology and immunohistochemistry

Methods were performed with minor modifications as previously described byEbeling et al.[38]. We collected the lung slices and either fixed them in 4% (v/v) buffered formalin, followed by paraffin embedding and sectioning or homogenized them for Western Blot analysis. E10 cells were harvested using a probing buffer (0,187 M Tris pH 6, 9; 6% SDS; 30% glycerine; 10 mM DTT and Bromophenolblue 40 mg/10 ml) for cell lysis and later loaded on a SDS-PAGE. AMJ2-C11 were separated from E10 by rinsing twice with PBS (Merck).

For Western Blot analysis total protein concentrations of homogenized lung slices were measured by Pierce BCA Protein Assay Kit (ThermoFisher Scientific) and total protein concentrations of E10 cell lysates were determined with the amidoschwarz protein assay earlier described by Dieckmann-Schuppert and Schnittler [39]. SDS-PAGE and Western Blot were performed as accurately described by Linge et al. [40]. After blocking with TBS-T buffer (137 mM NaCl,; 2, 7 mM KCl; 20 mM Tris-HCl pH 7.4; 0,05% Tween20) including 5% (w/v) dried non-fat powdered milk (Carl Roth GmbH, Karlsruhe, Germany) blots were incubated with the primary antibodies listed in Table 1. We used following secondary antibodies: donkey anti-rabbit IgG, HRP-linked F(ab)2 fragment (GE Healthcare, Chalfont St Giles, Buckinghamshire, United Kingdom), horse anti-mouse IgG, HRP-linked antibody (Cell Signaling, Danvers, MA, USA), and goat anti-Syrian hamster IgG-HRP (Santa Cruz, Dallas, TX, USA). Chemiluminescent signal was detected with Image Reader LAS-3000 (Fujifilm, Tokio, Japan) by following manufacture’s guidelines of Immobilon Western Chemiluminescent HRP Substrate (Merck). Quantification was done with ImageJ 1.43u free software (Wayne Rasband, National Institutes of Health, Bethesda, MD, USA) and each lane was normalized to corresponding γ-Tubulin (γ-Tub).

**Table 1:** Primary antibodies used for Western Blot (WB) and immunohistochemistry (IHC).

**Antibody**	**Dilution used for**		**Host**	**Type**	**Supplier**
	**WB**	**IHC**			
anti-γ-Tubulin clone GTU-88	1:500	-	Mouse	Monoclonal	Sigma-Aldrich (St. Louis, MO, USA)
anti-caspase-3 (Asp175)	-	1:50	Rabbit	Polyclonal	Cell Signaling (Danvers, MA, USA)
anti-caveolin 1 (clone 2297)	1:500	-	Mouse	Monoclonal	BD Biosciences (Franklin Lakes, NJ, USA)
anti-caveolin 1 (D46G3)	-	1:1600	Rabbit	Monoclonal	Cell Signaling (Danvers, MA, USA)
anti-caveolin 2 (clone 65)	1:500	1:400	Mouse	Monoclonal	BD Biosciences (Franklin Lakes, NJ, USA)
anti-P2X7R	1:500	-	Rabbit	Polyclonal	Alomone Labs (Jerusalem, Israel)
anti-PKC-*β*1	1:400	1:200	Rabbit	Polyclonal	Santa Cruz (Dallas, TX, USA)
anti-Podoplanin (T1α)	-	1:100	Hamster	Monoclonal	Kindly provided by M. Williams (Boston, MA, USA)
anti-Podoplanin (T1α) (8.1.1)	1:1000	-	Hamster	Monoclonal	Santa Cruz (Dallas, TX, USA)

Conventional histology was done as described by Ebeling et al. [38] and Sirius Red staining of collagen and reticulin fibers was made as previously described by Kasper et al. [41]. Immunohistochemistry was performed according to Kasper and Fehrenbach [42] as described in detail by Ebeling et al. [38].

### ELISA

Cytokine release was measured using an ELISA against IL-6 and TNF-α. The supernatant of following experiments was used for analyses: (i) PCLS of wild type mice with and (ii) without macrophages as well as (iii) PCLS of *P2rx7^−/−^*mice with and (iv) without macrophages. We used an ELISA kit from Affymetrix (Ready-SET-Go!—Santa Clara, CA, USA) following manufacture’s guidelines.

### FACS

To evaluate the viability of AMJ2-C11 as they interfere with nano-silica we used a calcein / Annexin V staining for FACS analysis. Living cells take up the non-fluorescent calcein-AM (ThermoFisher Scientific) and hydrolyses it to calcein, a strong fluorescent compound, by intracellular esterases. Dead cells were stained with Annexin V (BD Biosciences), a cellular protein binding phosphatidylserine and phosphatidylethenolamine which are only surfaced on the cell during apoptotic cell death.

Cells were collected and centrifuged, thereafter the medium was discarded. We rinsed the cells twice with PBS to eliminate medium remains, discarded the supernatant each time and stained with 100µl Annexin Binding buffer containing 1:4000 Calcein in DMSO and 1:200 Annexin V in Annexin Binding Buffer. After 15 minutes of incubation in darkness, 400 µl Annexin Binding buffer were added to each probe. After vortexing analysis was performed using FACSCalibur (BD Biosciences) following manufacture’s guidelines. Measurement was conducted with CellQuest 3.3 (BD Biosciences) till 10.000 cells were counted.

### Statistical analysis

Results are presented as means ± standard error of the mean (SEM). Statistical analysis was performed pretesting for a Gaussian distribution with Shapiro-Wilk-Test followed by a two-tailed student t test. Statistical analysis was done with GraphPad Prism 5.03 software (GraphPad Software, San Diego, CA, USA). The following significance levels were used:

* p ≤ 0.05; ** p ≤ 0.01. A minimum of three independent experiments were done.

## Results

### Treatment of PCLS with nano-silica as a model for early lung inflammation

First we assessed three markers for cell damage, apoptosis and repair in precision-cut lung slices: caspase 3 (Cc3), T1α and collagen deposition. T1α is an AEC I specific protein and early marker of lung injury [43]. Second we tested four proteins of AEC I involved in membrane integrity and cell signaling pathways: Cav-1 and caveolin-2 (Cav-2), two membrane proteins forming caveolae important for cell signaling, P2X7R, an AEC I specific purinergic receptor and protein kinase C-*β*1 (PKC-*β*1), a downstream signaling protein of P2X-receptors [44].

**Figure 1:**
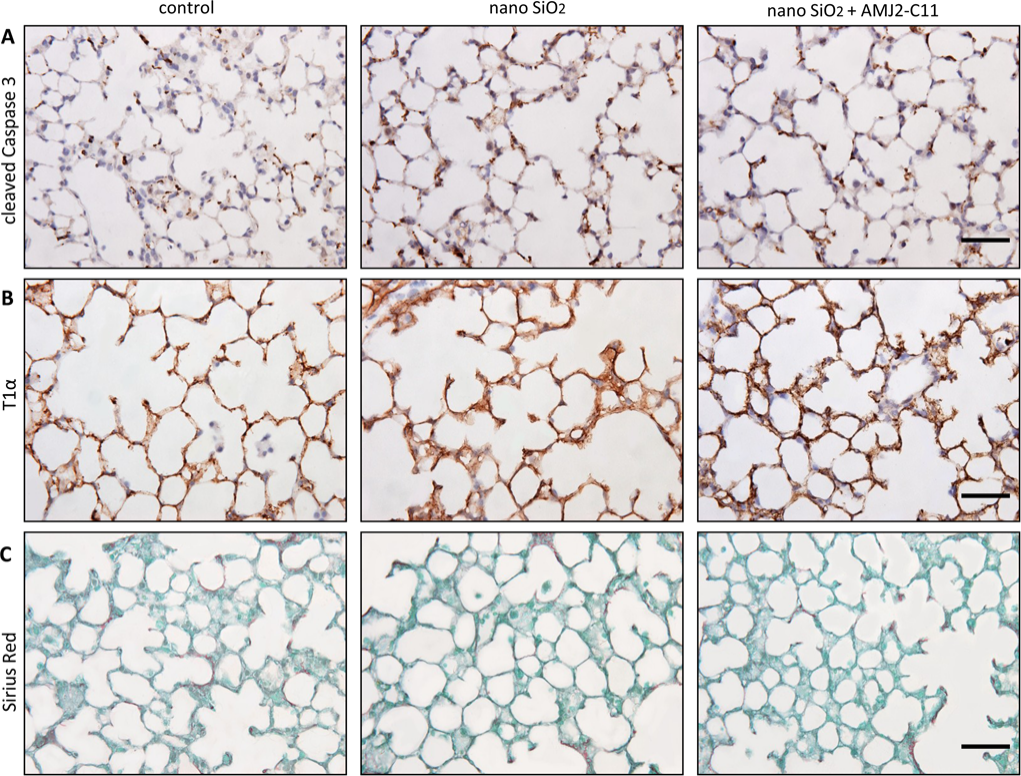
Evaluation of cell damage by nano-silica and macrophages in the PCLS of wild type mice. Immunohistochemical staining of Cc3 (A) and T1α (B), as well as Sirius Red staining (C) of representative untreated lung slices, slices treated with 1600 µg/ml silica and slices treated with 1600 µg/ml silica and co-cultured with AMJ2-C11 macrophages after 72 hours. Scale bar = 50 µm.

**Figure 2:**
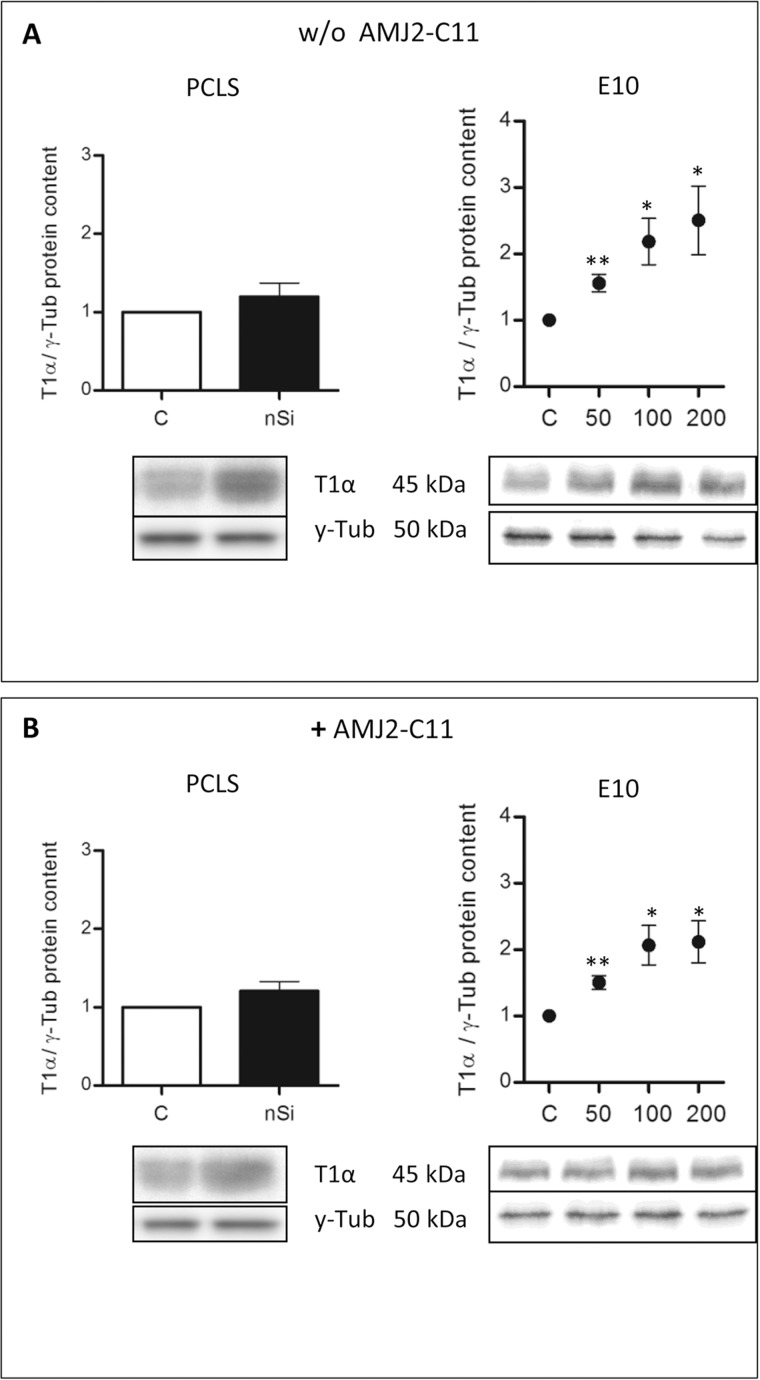
Protein content of T1α in the PCLS of wild type mice and the alveolar epithelial cell line E10. Immunoblots of PCLS and E10 experiments without (A) and with AMJ2-C11 macrophages (B). For PCLS the concentration of nano-silica was 1600 µg/ml and for E10 experiments nano-silica concentrations ranging from 50 to 200 µg/cm^2^ flask area were used. Data of each silica group was normalized to the control. Incubation time of PCLS was 72 hours and for E10 experiments 48 hours. γ-Tub is the loading control, n = 6–7.

We found an increase in the protein level of Cc3 (Fig. 1A) and T1α (Fig. 1B) indicated by immunohistochemical staining, but no differences in collagen deposition with Sirius Red staining (Fig. 1C) after incubation of the lung slices with 1600 µg/ml nano-silica for 72 hours. Immunocytochemical evaluation of T1α showed a protein level increase to 120% (Fig. 2A).

**Figure 3:**
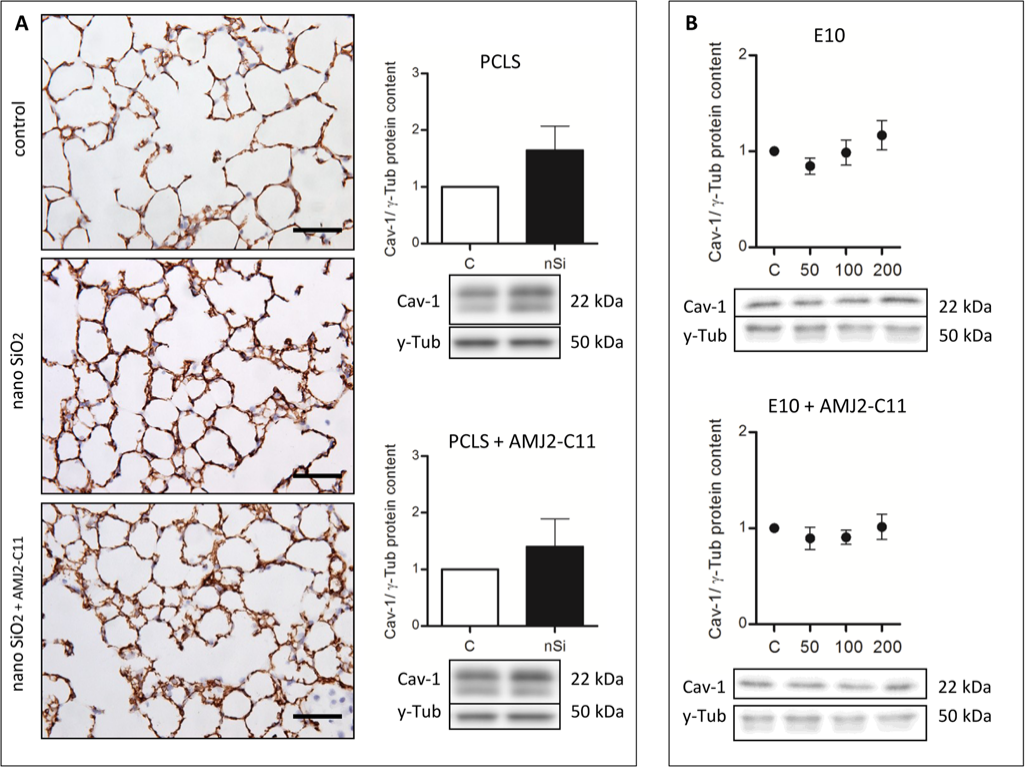
Cav-1 immunohistochemical staining and protein content of wild type mice PCLS and E10. (A) Immunohistochemical staining of Cav-1 of representative untreated lung slices, slices treated with 1600 µg/ml silica and slices treated with 1600 µg/ml silica and co-cultured with AMJ2-C11 macrophages as well as Western Blot analysis of PCLS. Data was normalized to the control. PCLS was incubated for 72 hours. (B) Immunoblots of E10 experiments without and with AMJ2-C11 macrophages. For E10 experiments nano-silica concentrations ranging from 50 to 200 µg/cm^2^ flask area and an incubation time of 48 hours were used. Each silica concentration was normalized to the control. γ-Tub is the loading control, n = 6–7. Scale bar = 50 µm.

**Figure 4:**
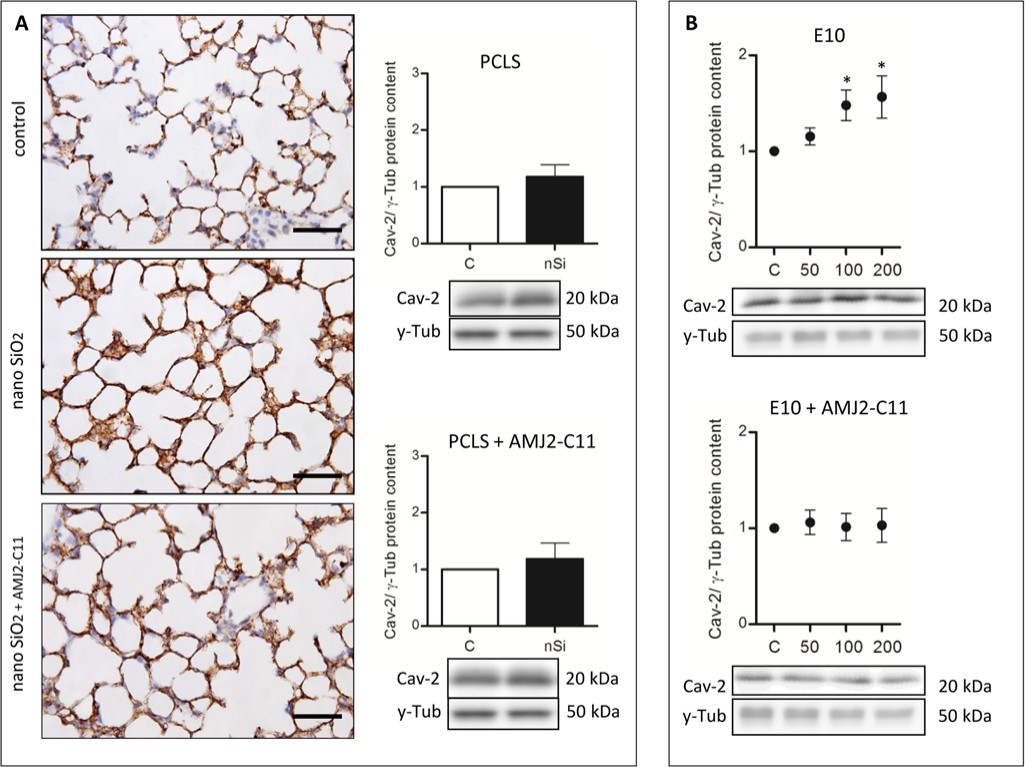
Cav-2 immunohistochemical staining and protein content of wild type mice PCLS and E10. (A) Immunohistochemical staining of Cav-2 of representative untreated lung slices, slices treated with 1600 µg/ml silica and slices treated with 1600 µg/ml silica and co-cultured with AMJ2-C11 macrophages as well as Western Blot analysis of PCLS. Data was normalized to the control. PCLS was incubated for 72 hours. (B) Immunoblots of E10 experiments without and with AMJ2-C11 macrophages. For E10 experiments nano-silica concentrations ranging from 50 to 200 µg/cm^2^ flask area and an incubation time of 48 hours were used. Each silica concentration was normalized to the control. γ-Tub is the loading control, n = 6–7. Scale bar = 50 µm.

**Figure 5:**
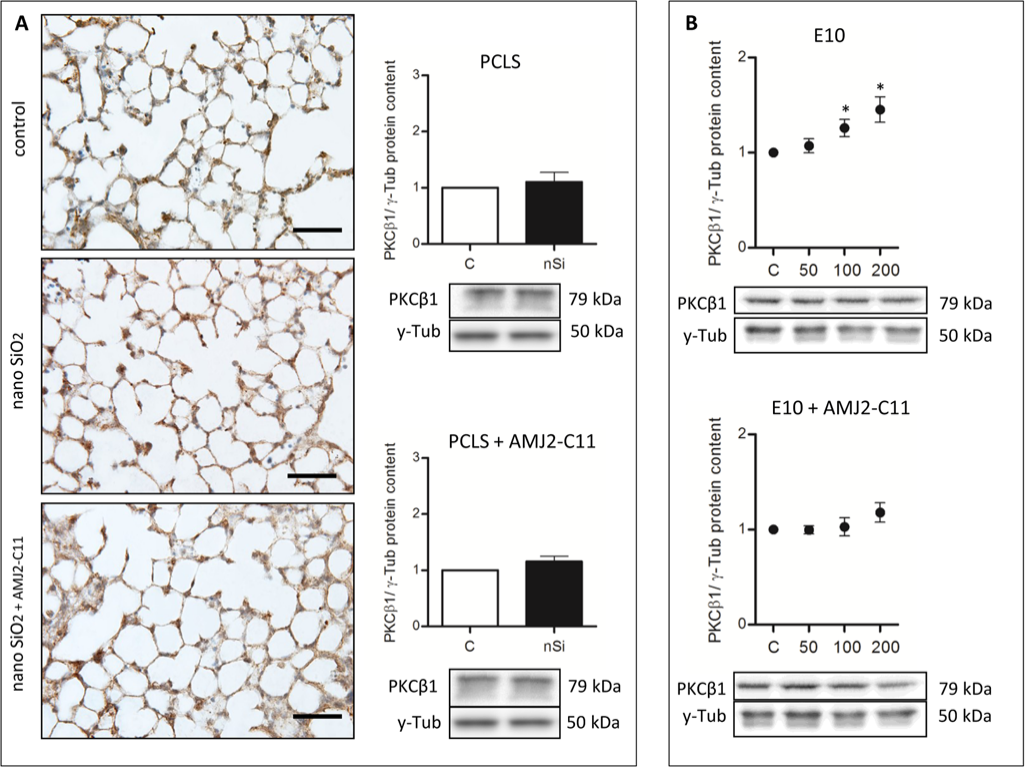
PKC-*β*1 immunohistochemical staining and protein content of wild type mice PCLS and E10. (A) Immunohistochemical staining of PKC-*β*1 of representative untreated lung slices, slices treated with 1600 µg/ml silica and slices treated with 1600 µg/ml silica and co-cultured with AMJ2-C11 macrophages as well as Western Blot analysis of PCLS. Data was normalized to the control. PCLS was incubated for 72 hours. (B) Immunoblots of E10 experiments without and with AMJ2-C11 macrophages. For E10 experiments nano-silica concentrations ranging from 50 to 200 µg/cm^2^ flask area and an incubation time of 48 hours were used. Each silica concentration was normalized to the control. γ-Tub is the loading control, n = 6–7. Scale bar = 50 µm.

**Figure 6:**
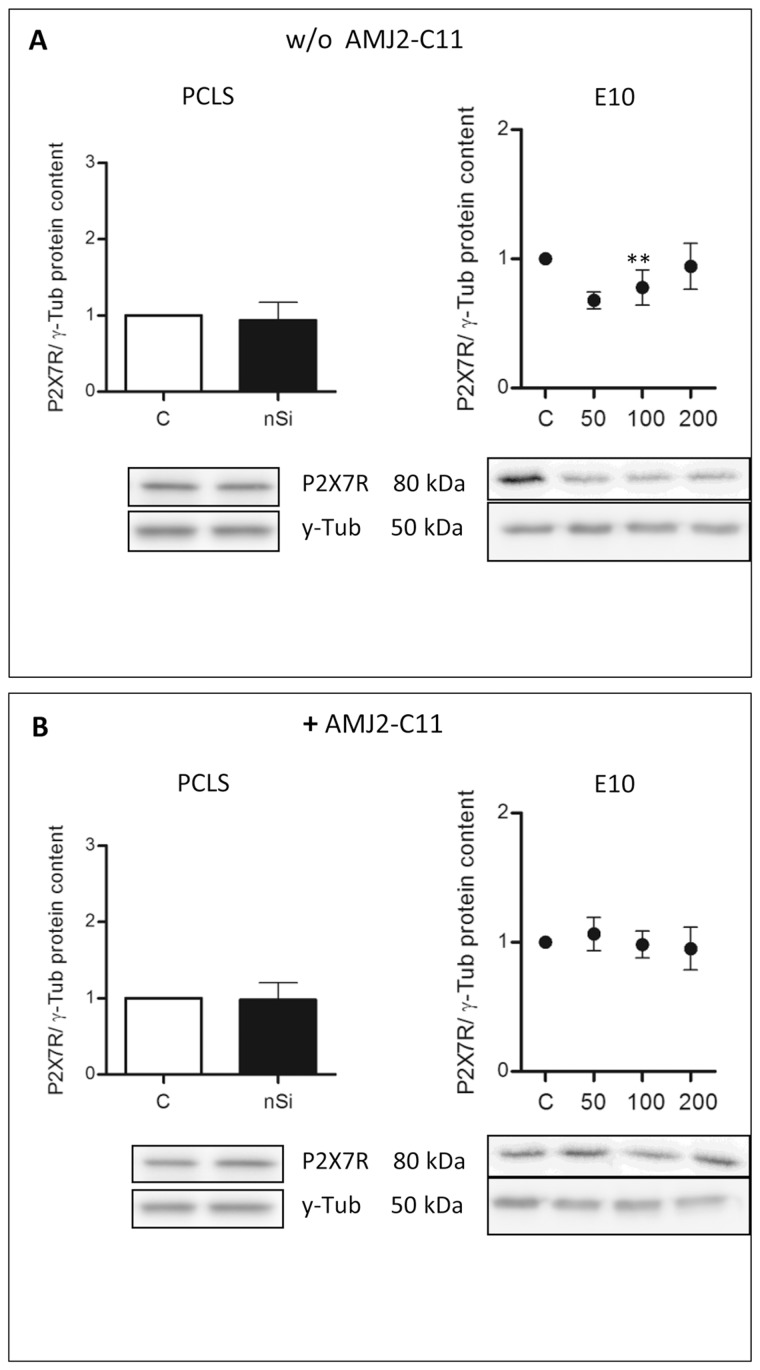
Protein content of P2X7R in the PCLS of wild type mice and E10. Immunoblots of PCLS and E10 experiments without (A) and with AMJ2-C11 macrophages (B) after 72 hours incubation. For PCLS the concentration of nano-silica was 1600 µg/ml and for E10 experiments nano-silica concentrations ranging from 50 to 200 µg/cm^2^ flask area and an incubation time of 48 hours were used. Each silica concentration was normalized to the control. γ-Tub is the loading control, n = 6–7.

Western Blot analysis of the AEC I proteins showed an increase to 164% for Cav-1 (Fig. 3A), 118% for Cav-2 (Fig. 4A) and 110% for PKC-*β*1 (Fig. 5A), whereas P2X7R decreased to 94% (Fig. 6A).

### Effects of nano-silica on the cell line E10

To verify the results from the PCLS we analysed the influence of nano-silica on the alveolar epithelial cell line E10. Also we determined the dose-dependency of cytotoxicity by using 3 concentrations of nano-silica: 50, 100 and 200 µg/cm^2^.

The treatment with nano-silica showed a high increase of the T1α protein level ranging from 156% at a nano-silica concentration of 50 μg/cm^2^ up to 250% at 200 μg/cm^2^ (Fig. 2A). Protein level of Cav-1 showed a decrease to 85% with 50 μg/cm^2^ nano-silica, but with an increasing concentration of nano-silica (c_nSi_) Cav-1 protein level also increased up to 117% (Fig. 3B). Protein content of Cav-2 also increased ranging from 115% up to 157% (Fig. 4B). The protein level of PKC-*β*1 rose with raising c_nSi_ from 107% up to 145% (Fig. 5B). P2X7R on the other hand showed a decrease in the protein level to 68%, but rose with increasing c_nSi_ nearly up to the control again (Fig. 6A).

### Influence of macrophages on PCLS and the cell line E10

The lung interfaces with blood and air and is therefore exposed to a vast array of inhaled antigens and particulate matter and thus endangered of e.g. infections. To protect the organism from microbiological invasion the lung has a very high concentration of alveolar macrophages. Our present in vitro PCLS model lacks residual alveolar macrophages and other inflammatory cells of the circulation due to the artificial explant conditions.

To investigate the influence of macrophages we used a co-culture with AMJ2-C11 both in the cell culture with E10 and in the PCLS. The comparison of the PCLS experiments (with or without macrophages) showed some differences:

Apoptosis was lower with macrophages, indicated by a weaker increase of Cc3 (Fig. 1A).

Collagen deposition, as indicated by Sirius Red staining, was on the same level as without macrophages (Fig. 1C).

The increase of the Cav-1 protein level was by 20% weaker with macrophages than without (Fig. 3A), whereas T1α, Cav-2 and PKC-*β*1 showed a similar increase in the protein content (Fig. 2B, 4A and 5A). The protein content of P2X7R was at the same level as in the PCLS without macrophages (Fig. 6B).

The co-culture of the cell line E10 with macrophages revealed some major changes in the protein levels. T1α showed an increase of protein content to 150–200%, but stagnated at 200 μg/cm^2^ nano-silica with 210% (Fig. 2B). The amount of Cav-1 was reduced to 89% with a little increasing at higher c_nSi_ up to 101% (Fig. 3B). Cav-2 was on the protein level of the control with all concentrations of nano-silica (Fig. 4B). The protein content of PKC-*β*1 increased to 103% at a concentration of 100 μg/cm^2^ nano-silica and to 117% at 200 μg/cm^2^ (Fig. 5B). The last protein, P2X7R, oscillated around the level of the control (Fig. 6B).

### Differences in the induction of lung inflammation between wild type mice and *P2rx7^−/−^*mice

In this experiment we evaluated the influence of the P2X7R in the early phase of inflammation. For this purpose we used *P2rx7^−/−^*mice in comparison to wild type mice.

Similar to the above described method we assessed the changes in the protein content of Cav-1, Cav-2, T1α and PKC-*β*1 with and without macrophages.

**Figure 7:**
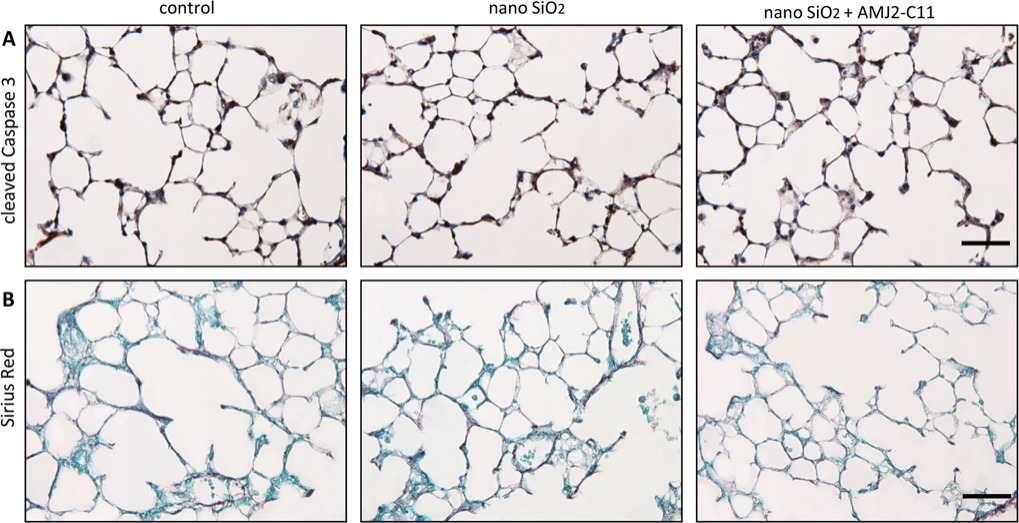
Evaluation of cell damage by nano-silica and macrophages in the PCLS of *P2rx7^−/−^* mice. Immunohistochemical staining of Cc3 (A) and Sirius Red staining (B) of untreated lung slices, slices treated with 1600 µg/ml silica and slices treated with 1600 µg/ml silica and co-cultured with AMJ2-C11 macrophages after 72 hours incubation. Scale bar = 50 µm.

Immunohistochemical staining of Cc3 showed a little increase of apoptosis after lung slice incubation with 1600 µg/ml nano-silica (Fig. 7A). We found no change in collagen deposition with Sirius Red staining (B).

**Figure 8:**
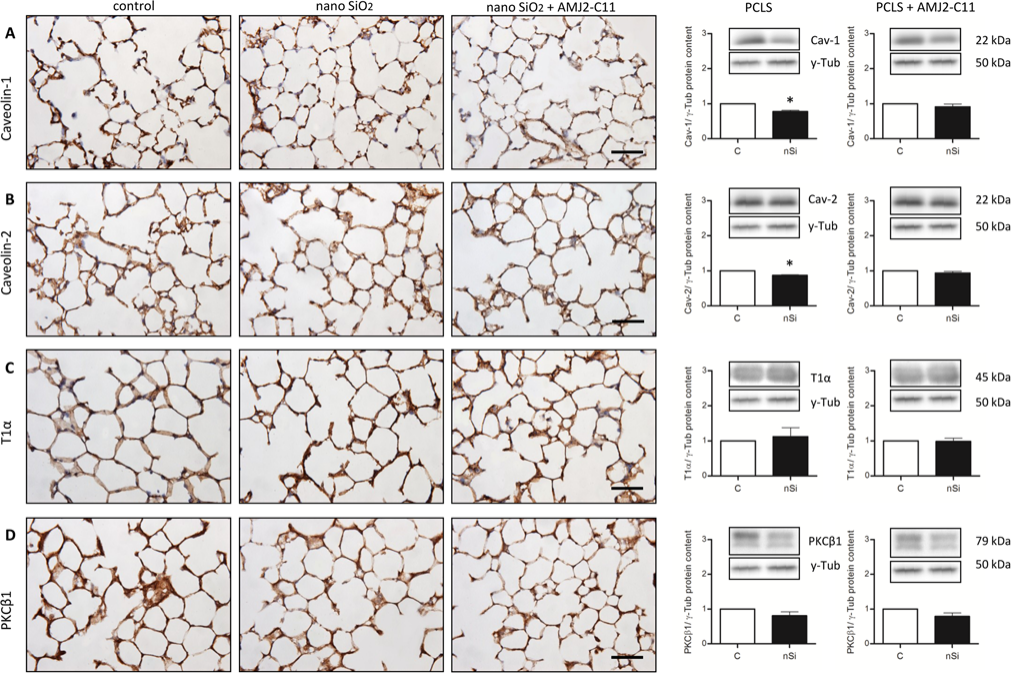
Immunohistochemical staining and protein content of Cav-1 (A), Cav-2 (B), T1α (C) and PKC-*β*1 (D) of untreated lung slices, slices treated with 1600 µg/ml silica and slices treated with 1600 µg/ml silica and co-cultured with AMJ2-C11 macrophages after 72 hours incubation. γ-Tub is the loading control, n = 6–7. Scale bar = 50 µm.

The protein levels of Cav-1 and Cav-2 were significantly decreased to 78% and 87% respectively after 72 hours incubation with 1600 µg/ml nano-silica without macrophages. PKC-*β*1 also decreased to 81%, whereas T1α showed an increase to 112% (Fig. 8). With macrophages all protein levels are decreased ranging from 98% for T1α down to 79% for PKC-*β*1 (Fig. 8).

### Secretion of TNF-α and IL-6 in the PCLS of wild type and *P2rx7^−/−^* mice

**Figure 9:**
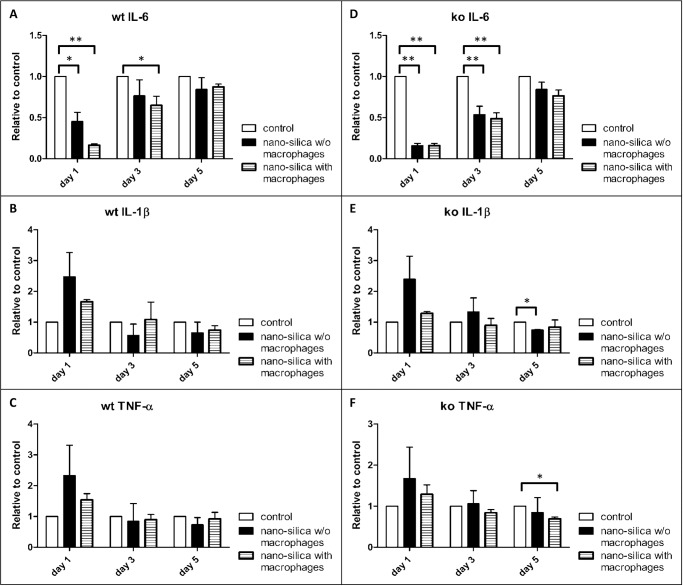
Measurement of IL-6 (A, D), TNF-α (B, E) and IL-1β (C, F) in the supernatant of the PCLS via ELISA. Compared are wild type (wt) and *P2rx7^−/−^* (ko) mice that were cultivated with and without (w/o) AMJ2-C11 macrophages with a nano-silica concentration of 1600 µg/ml. Data is presented in relation to the controls. The incubation time was 72 hours. n = 6–9.

We used for our experiments AMJ2-C11 macrophages, which secrete substantial amounts of IL-6 under stimulation with LPS, but not TNF-α [36]. To evaluate the influence of the macrophage produced IL-6, we measured the content of IL-6 and also the content of TNF-α in the supernatant of the PCLS after one, three and five days. We collected the supernatant of the PCLS of wild type and *P2rx7^−/−^* mice, each with and without macrophages as described under “Precision-cut lung slices and tissue culture”. The amount of IL-6 was significantly decreased in the supernatants of slices incubated with silica after one and three days compared to the controls. Remarkably addition of macrophages increased this effect. After five days the synthesis of IL-6 was restored and no differences could be detected compared to the controls. Similar effects were observed in *P2rx7^−/−^* mice (Fig. 9). However there is a difference between wild type and *P2rx7^−/−^* mice regarding the IL-6 content in the supernatant in the comparison of slices with and without macrophages after one day. In the wild type mice the addition of macrophages increased the effect of silica on the level of IL-6 whereas in the *P2rx7^−/−^* mice the macrophages had no further increasing effect.

The supernatant of slices incubated with silica showed an increase of TNF-α in wild type mice as well as in *P2rx7^−/−^* mice compared to the controls after one day. The addition of macrophages decreased this effect (Fig. 9). After three days no effect of silica or silica together with macrophages could be observed in both groups compared to the controls. Incubation with silica had also no effects on the level of TNF-α in both groups after five days whereas addition of macrophages moderately decreased the amount of TNF-α in the *P2rx7^−/−^* mice (Fig. 9).

The effects of silica and macrophages on the levels of IL-1β are quite similar to the effects on the amounts of TNF-α in wild type and in *P2rx7^−/−^* mice (Fig. 9).

### Uptake of nano-silica by AMJ2-C11

One explanation of the influence of macrophages on the development and ongoing of early lung inflammation and pulmonary fibrosis is their phagocytosis of silica particles [45]. Once inside the particles seem to kill the macrophages by generation of reactive oxygen species [9].

**Figure 10:**
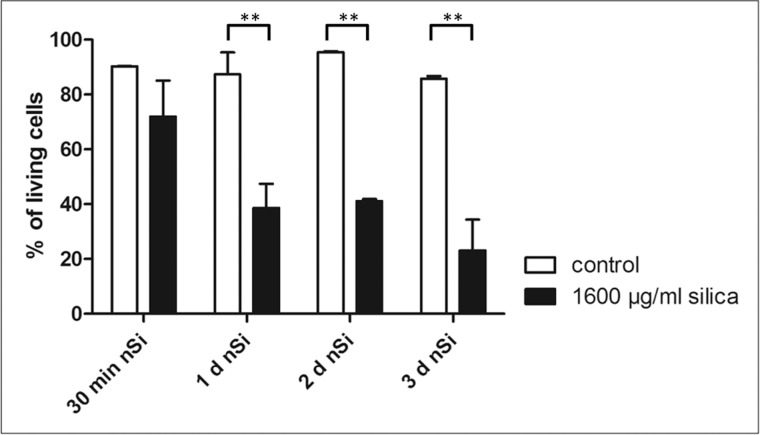
FACS analysis on the viability of AMJ2-C11 macrophages incubated with 1600 µg/ml nano-silica. The incubation times are 30 minutes, 24 hours, 48 hours and 72 hours. Data is presented in relation to the controls of each time point. n = 3.

To assess the time course of cell death after interaction with nano-silica we used a calcein / Annexin-V staining for flow cytometry analyses. We incubated the AMJ2-C11 with 1600 µg/ml nano-silica for 30 minutes, 24 hours, 48 hours and 72 hours. Independently of the point in time the controls showed approximately 90% living cells, whereas the macrophages incubated with silica showed a decreasing percentage of living cells. After 30 minutes incubation with silica vitality decreased to 72% and after 3 days the vitality was at 23% (Fig. 10).

## Discussion

Silica is well known to induce oxidative stress and to be responsible for the release of inflammatory mediators leading in many cases to the development of fibrotic respiratory diseases [19]. There is evidence that the inhalation of such substances can induce the formation of the inflammosome complex, which involves the activation of caspase-1 and the release of proinflammatory cytokines IL-1β and IL-18 [46]. The involvement of purinergic signaling via P2X7 receptor in macrophages exposed to silica nanoparticles has been demonstrated by [47].

The current *in vitro* PCLS model revealed several differences to experimental animal models of chronic injury, where during the course of disease, for example in pulmonary fibrosis, a number of AEC I specific proteins are strongly diminished: T1α [48], ICAM-1 [49], aquaporin-5 [50], RAGE [51] and Cav-1 [52].

First, the exposure of immortal AEC I—like E10 cells as well as of PCLS to nano-silica particles induced an early increase of T1α, Cav-1 and -2, as well as PKC-*ß*1 within 72 hours. Similar *in vitro* induction of Cav-1 and T1α was obtained *in vitro* after exposure of alveolar epithelial cells to bleomycin [40]. The reason for this apparent discrepancy is not yet clear. Data from early time points of protein expression in alveolar epithelial cells of animal models after exposure with toxic and other agents are missing.

Second, the co-culture of lung slices with an immortal cell line maintaining properties of alveolar macrophages revealed unexpected alterations in the level of expression of acute lung cell markers in both directions: up- and downregulation. From these data we suggest that the co-culture of inflammatory cells with PCLS may provide a new tool for the study of the contribution of the systemic aspect (e.g. circulating inflammatory and immune cells) and its relationship to the resident cells of the lung parenchyma during inflammatory and other pathologic conditions.

Third, the comparison of the induction of lung inflammation in wild type mice showed an increased protein level of Cav-1 and -2 in contrast to the decreased protein levels of Cav-1 and -2 in *P2rx7^−/−^* mice. Nano-silica particles induced an inflammatory response such as increase of TNF-a and Il-1β after one day in wild type and *P2rx7^−/−^* mice. This response disappeared after prolonged incubation for three and five days. This resembles to a stress response like process.

*P2rx7^−/−^* mice showed a slight decreased Cav-1 and Cav -2 protein content. These effects have to be discussed in terms of the strong functional interaction of P2X7R isoforms with Cav-1 in caveolae, microdomains of AEC I. Previous studies of *P2rx7^−/−^* mice have shown a decreased Cav-1 protein level in lung epithelial type I cells [53]. Also Cav-1 downregulation in the E10 cell line resulted in a decreased P2X7R protein level [53].

These effects are unchanged in the presence of alveolar macrophages. These macrophages may contribute to early lung damage because of their secretion of pro-inflammatory substances like IL-6 [54]. Induction of cytokine release seems to depend on a damaging agent like silica or LPS and cell-cell interactions. [14] showed in a macrophage mono-culture after 24 hours incubation with nano-silica no significant release of IL-6 and IL-8, but high amounts in a co-culture with an alveolar epithelial cell line. Our macrophage cell line failed to produce higher levels of IL-6, TNF-a and IL-1β. Maybe this was due to a very high silica concentration that killed most macrophages as shown in our FACS experiment with the same nano-silica concentration as used in the PCLS experiments. Beside their potential pro-inflammatory effects, macrophages play a critical role for the pulmonary clearance like the transport of particulate reagents e.g. silica outside the alveoli and thus reducing the silica lung burden. In macrophages which phagocytosed nano-silica, agglomerated silica particles can be detected around the nucleus [14]. After intratracheal instillation of nano-silica these macrophages are primarily residing within airways and alveoli, but at subsequent times they can be found in the peribronchial, perivascular, interstitial, and subpleural connective tissues [55]. Together with our findings, that nano-silica phagocytosing macrophages are heavily damaged but still produce inflammatory mediators, we suggest that their migration into the interstitium is a central part for interstitial damage possibly leading to subsequent misrepair.
